# 在线固相萃取-液相色谱-线性离子阱质谱法同时检测尿液和血液中51种吲唑类合成大麻素

**DOI:** 10.3724/SP.J.1123.2024.02008

**Published:** 2025-02-08

**Authors:** Xuan LUO, Jun ZHANG, Dingji ZHU, Kejian HUANG, Ning YANG, Xiaofeng LIU, Qiulian LUO

**Affiliations:** 1.广西大学化学化工学院,广西南宁530004; 1. School of Chemistry and Chemical Engineering, Guangxi University, Nanning 530004, China; 2.广西壮族自治区职业病防治研究院,广西南宁530021; 2. Institute of Forensic Science, Public Security Department of Guangxi Zhuang Autonomous Region, Nanning 530021, China; 3.广西壮族自治区公安厅物证鉴定中心,广西南宁530012; 3. Institute of Forensic Science, Public Security Department of Guangxi Zhuang Autonomous Region, Nanning 500012, China; 4.广西大学广西高校应用化学技术与资源开发重点实验室,广西南宁530004; 4. Guangxi Colleges and Universities Key Laboratory of Applied Chemistry Technology and Resource Development, Guangxi University, Nanning 530004, China

**Keywords:** 合成大麻素, 吲唑类, 尿液, 血液, 在线固相萃取, 液相色谱-线性离子阱质谱, synthetic cannabinoids (SCs), indazole-type, urine, blood, online solid-phase extraction (online SPE), liquid chromatography-linear ion trap-mass spectrometry (LC-LIT-MS)

## Abstract

合成大麻素(SCs)是数量最多的一类新精神活性物质(NPS)。我国于2021年7月起对SCs实行了整类列管。然后,经过结构修饰后产生的新型SCs仍然层出不穷,给法庭科学实验室检测分析带来巨大挑战。因此,亟需构建高效、绿色和自动化的分析检测方法为真实案件样品的准确筛查提供技术支持。同时,吲唑类SCs因药效更强,自2013年后数量急剧增加,是SCs中需要重点关注的一个亚类,也是法庭科学实验室检测分析SCs的主要类别。本文开发了在线固相萃取前处理技术结合液相色谱-线性离子阱质谱(online SPE/LC-LIT-MS)从人体尿液和血液中检测51种吲唑类SCs的分析方法。将加入了51种吲唑类SCs混合标准溶液和内标溶液的人血液或尿液试样经乙腈沉淀蛋白后,用含0.1%甲酸的10 mmol/L醋酸铵溶液(pH=4.8)稀释,过滤后直接进样;以含0.1%甲酸(v/v)的乙腈-含0.1%甲酸的10 mmol/L醋酸铵溶液为流动相进行分析。在全扫描模式下,选择目标分析物的分子离子([M+H]^+^)和保留时间监测其二级离子,共实现了51种吲唑类SCs的定量分析;结合色谱和多级质谱数据也实现了的定性筛查(包括5组同分异构体)。各分析物检出限为0.02~1 ng/mL,定量限为0.04~4 ng/mL(尿液)和0.1~4 ng/mL(血液)。采用线性拟合时(权重因子1/*x*),各分析物在各自的线性范围内线性关系良好。同时在定量限、低、中、高4个水平下考察了方法的回收率和精密度,回收率为83.47%~116.95%,精密度为0.58%~13.79%。本文方法通过阀切换在动态模式下实现样品的提取、富集和分析,不仅操作简单,还实现了样品的自动化和高通量分析;同时,具有较好的灵敏度和更宽的适用范围,为实际案件中SCs的快速筛查和定量分析提供了科学依据和技术支持。

## Abstract (en)

To evade legal controls, new psychoactive substances (NPS), which have been designed as substitutes for traditional and synthetic drugs, are gradually dominating the drug market. Synthetic cannabinoids (SCs), which account for the majority of NPS, are rapidly being derivatized; consequently, controlling increasing abuse by merely listing individual compounds is difficult. Therefore, China has included the entire SC category of SCs listed as legal controlled substances since July 1, 2021. However, new SCs obtained through structural modification are still appearing and pose significant analytical challenges for forensic laboratories. Therefore, an efficient, green, and automated detection method is urgently required to provide technical support for the accurate screening actual samples. Meanwhile, the number of indazole-type SCs has increased sharply since 2013, which is ascribable to their stronger psychoactive effects. Indeed, forensic laboratories mainly analyze this key SC subclass. Therefore, in this study, we developed a new method for analyzing 51 indazole-type SCs in human urine and blood, which involves online solid-phase extraction (online SPE) as the preprocessing technology, with analysis performed using liquid chromatography-linear ion trap mass spectrometry. Deproteinization was achieved by adding acetonitrile, with dilution performed using 10 mmol/L ammonium acetate solution (pH 4.8) containing 0.1% formic acid. Samples were then analyzed directly using acetonitrile-10 mmol/L ammonium acetate aqueous solution (containing 0.1% formic acid) as the mobile phase. The mass-to-charge ratios of protonated molecular ions ([M+H]^+^) in the mass spectra acquired in full-scan mode, and the retention times in the chromatograms of the analytes were selected with the aim of monitoring the MS^2^ ions of the various compounds. Characteristic fragment ions of the various SC structures were summarized, with five groups of isomers, each containing ten compounds, successfully distinguished using multistage mass spectrometry and their retention times. Multistage MS was used to qualitatively screen 51 indazole-type SCs, which were then quantitatively analyzed using MS^2^ ion pairs (as quantitative ion pairs). The analytes exhibited limits of detection (LODs) of 0.02-1 ng/mL, with limits of quantification (LOQs) of 0.04-4 and 0.1-4 ng/mL in urine and blood, respectively. Linear fitting (weighting factor 1/*x*) revealed good linearity for each analyte within its respective linear range, with correlation coefficients (*R*^2^) greater than 0.99 in both urine and blood. The validity of the analytical method was tested by determining precision and spiked recovery values (*n*=6). Recoveries of 83.47%-116.95% were obtained at LOQ levels, with precisions of 2.29%-13.40%. In addition, recoveries of 86.63%-113.38% and precisions of 0.58%-13.79% were obtained at low, medium, and high levels. The method described herein is not only easy to operate but also can be automated. Indeed, high-throughput sample analysis was achieved when sample extraction, enrichment, and analysis were performed in dynamic mode through valve switching. Meanwhile, the method exhibited good sensitivity and is applicable to a wider range of compounds than those previously reported; it also provides a scientific basis and technical support for the rapid screening and quantitative analysis of SCs in actual relevant cases.

新精神活性物质(NPS)是通过对现有受控物质进行结构修饰而衍生出的新型毒品^[1⇓-3]^,截至2023年11月,全球范围内已报道1230种NPS,其中近1/3为合成大麻素(SCs),是数量最多的一类NPS^[4]^。2008年,第一个氨基烷基吲哚类SCs——1-戊基-3-(1-萘甲酰基)吲哚(又称JWH-018)被报道,自此毒品市场上涌现出大量围绕这一结构而产生的SCs类似物^[5]^。然而,2013年具有吲哚结构的SCs数量减少,而具有吲唑结构的SCs数量急剧增加。基于药理学特性的研究表明,吲唑结构的SCs较吲哚结构的SCs更能增强SCs的效力,因此经过结构修饰后产生的新结构吲唑类SCs不断出现在毒品市场上^[6⇓-8]^。为逃避法律的监管和打击,犯罪分子对SCs结构进行修饰得到类似物的速率非常快,因此我国于2021年7月按照结构对SCs进行整类列管,成为首个整类列管SCs的国家^[9⇓-11]^。但2022年,在欧洲发现的23种首次被报道的SCs中,竟有多达20种是专门针对我国整类列管SCs而进行的结构修饰,其结构不在我国法律针对SCs整类列管的特征结构范畴内^[6]^。此外,不断出现的新结构SCs也给法庭科学实验室检测工作带来了巨大挑战^[5,12⇓-14]^。

通常,在摄入SCs后,药物首先被吸收并进入血液,经过一系列的体内代谢过程后部分经尿液排出。因此,血液中常可检测到母体药物,而尿液中母体药物浓度较低^[15]^。尽管如此,尿液样本的采集既简单又无创伤,依然是许多实验室首选的标本^[16,17]^。

目前,液相色谱-质谱法(LC-MS)凭借高灵敏度和高选择性已广泛应用于尿液^[17⇓⇓-20]^、血液^[18,21⇓⇓-24]^、口腔液^[25⇓-27]^和毛发^[28]^等多种生物基质中SCs的检测。目前大多数生物基质中SCs的分析方法均采用液相色谱-串联质谱(LC-MS/MS)技术,其最大的优势是检出限低,可低至约0.01 ng/mL^[17,23,24,29⇓-31]^,但LC-MS/MS在面对同分异构体的甄别时仍有不足。与此同时,采用LC-MS分析复杂生物基质中的微量药物时,选择高效的前处理方法十分重要^[15]^。固相萃取(SPE)因其可以同时实现样品净化和富集而受到青睐,Luo等^[32]^基于SPE的富集作用鉴定出了尿液中数量最多的2-氟胺酮代谢物;王冠翔等^[30]^和古锟山等^[33]^采用蛋白质沉淀和SPE两种前处理方法分别分析了血液和尿液中的SCs,结果均显示采用SPE为前处理手段时可以获取更低的检出限。在线固相萃取(online SPE)相比于传统SPE更高效,且具有更高的通量。Online SPE不仅保留了SPE原本的优势,而且自动化的样品处理减少了分析时间和潜在的样品污染和损失;可重复使用的萃取小柱极大地提高了分析通量,降低了分析成本。目前,在线固相萃取-液相色谱-质谱(online SPE-LC-MS)的检测方法在食品^[34,35]^和水污染^[36,37]^等领域已有所应用,本课题组前期也在法庭科学领域使用online SPE作为前处理方法开发了一系列毒物分析方法^[38⇓⇓-41]^,但其在SCs检测方面的应用仍有限。

基于以上考虑,本文旨在建立一种基于在线固相萃取-液相色谱-线性离子阱质谱(online SPE-LC-LIT-MS)的分析方法,用于快速筛查和定量分析血液和尿液中51种吲唑类SCs。这一分析方法的建立将为相关实际案件中SCs的分析提供技术支持,以应对不断变化的SCs市场。

## 1 实验部分

### 1.1 仪器与试剂

在线固相萃取/液相色谱-线性离子阱质谱联用仪(美国赛默飞世尔科技有限公司),其中包括Ultimate 3000双三元梯度液相色谱、LTQ-XL线性离子阱质谱仪,并配备Xcalibur数据处理系统;XS205DU分析天平(瑞士梅特勒托利多公司); MS3混合器(德国IKA公司); AP 300全自动液体样品处理工作站(中国睿科基团股份有限公司)。

实验所用HPLC级乙腈、甲酸、醋酸铵、甲醇、丙酮和异丙醇购自美国赛默飞世尔科技有限公司;51种吲唑类SCs标准品(基本信息如图1所示)和内标化合物*N*-(1-金刚烷基)-1-戊基-1*H*-吲唑-3-甲酰胺-d_9_(APINACA-d_9_)均购自上海原思标物科技有限公司。根据欧洲毒品和吸毒成瘾监测中心(EMCDDA)提出的不同SCs结构模型^[42⇓-44]^,按本文所研究的51种吲唑类SCs在结构中取代基的差异将其分为5类,即(a)1-烃基-2-氨基-2-羰基乙基类、(b)1-烃基-2-甲氧基-2-羰基乙基或1-烃基-2-乙氧基-2-羰基乙基类、(c)2-苯基-异丙基类、(d)*α*-萘基和(e)金刚烷基类。

**图 1:**
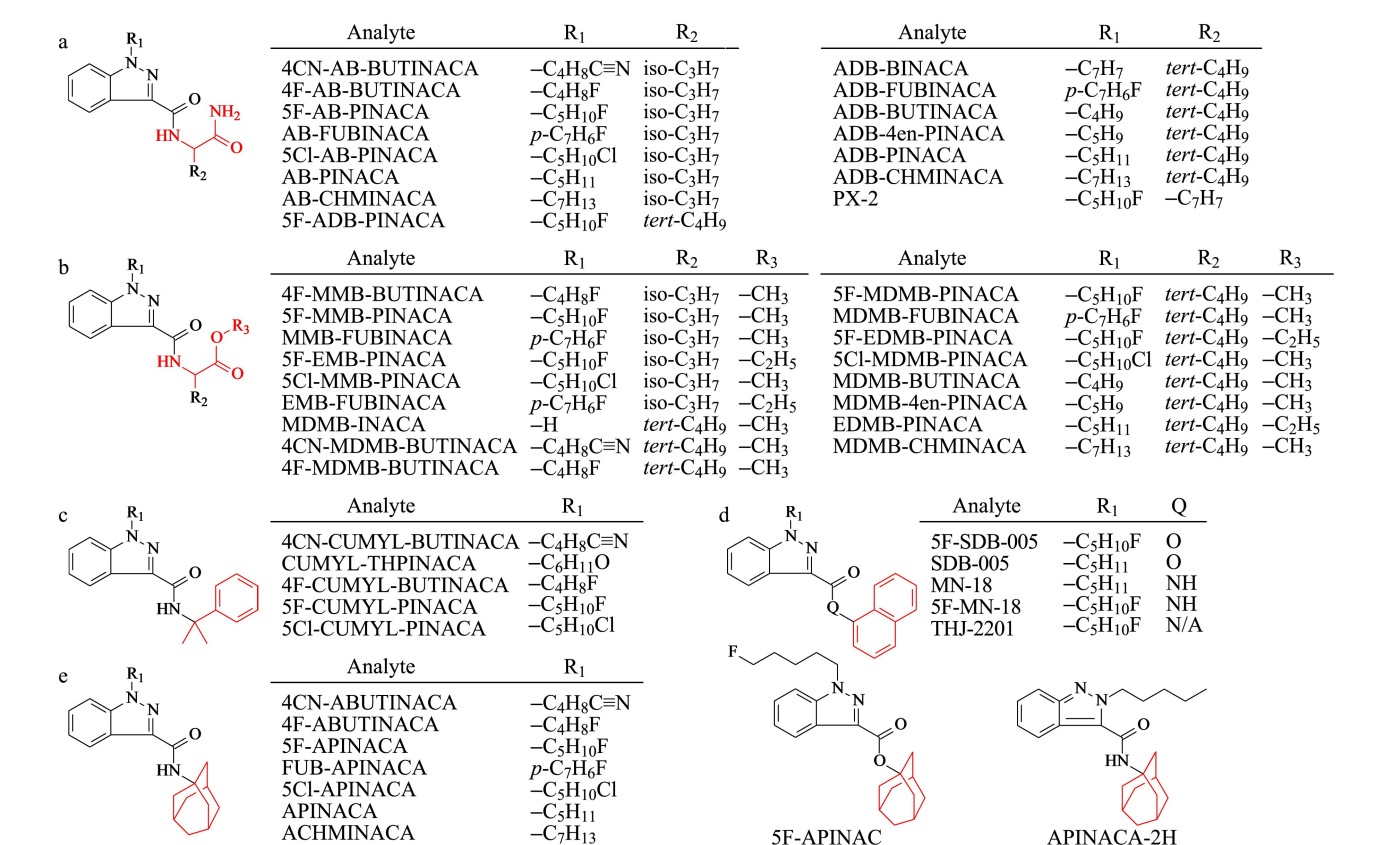
合成大麻素的基本信息

人体尿液和血液样本均由广西壮族自治区公安厅物证鉴定中心提供,健康志愿者均签署知情同意书。

### 1.2 标准溶液的配制

分别称取51种吲唑类SCs标准品1.5~4 mg,用乙腈溶解后配制成2 mg/mL的单一标准储备液。各移取25 μL 51种吲唑类SCs单一标准储备液,混合后用乙腈稀释至5 mL,配制成10 μg/mL的混合标准溶液。采用全自动液体样品处理工作站,将10 μg/mL的混合标准溶液用乙腈稀释,分别配制成5000、2000、1000、500、200、100、50、20、10、5、2、1和0.5 ng/mL的系列混合标准溶液,备用。采用相同方式配制含APINACA-d_9_质量浓度为2 mg/mL的标准储备液。分两次稀释配制成200 ng/mL的内标(IS)溶液,备用。

### 1.3 样品溶液的制备

准确移取1.0 mL空白血液或尿液,加入20 μL系列混合标准溶液和20 μL IS溶液,再加入2.96 mL乙腈沉淀蛋白,振荡1 min后,离心10 min,移取2.0 mL上清液,加入1.75 mL含0.1%甲酸(v/v)的醋酸铵溶液(10 mmol/L)进行稀释。再次离心后取上清液经0.22 μm有机滤膜过滤,滤液备用。

### 1.4 仪器条件

Online SPE: online SPE柱为Oasis HLB(20 mm×2.1 mm, 15 μm);流动相为水和甲醇;进样量为150 μL;梯度洗脱程序见表1,其中前4 min以水作为流动相,SPE柱在线富集,4 min时通过阀切换,将SPE柱与分析色谱柱相连,8 min后断开连接,分别用甲醇、水对SPE柱进行活化,为下一个样品分析做准备。

**表1:** Online SPE梯度洗脱条件

Time/ min	Flow rate/ (mL/min)	*φ*(Water)/ %	*φ*(Methanol)/ %
0	0.4	100	0
4	0.4	100	0
5	0.4	0	100
8	2	0	100
10	2	0	100
10.1	2	100	0
12	2	100	0
12.1	0.4	100	0

液相色谱分离:分析柱为ZORBAX Eclipse Plus C_18_(100 mm×2.1 mm, 1.8 μm);流动相A为含0.1%甲酸(v/v)的10 mmol醋酸铵溶液,流动相B为含0.1%甲酸(v/v)的乙腈;流速为0.3 mL/min;柱温40 ℃;梯度洗脱程序见表2。

**表2:** 液相色谱梯度洗脱条件

Time/min	*φ*(A)/%	*φ*(B)/%
0	70	30
4	70	30
15	10	90
18	10	90
20	70	30

A: 10 mmol/L ammonium acetate containing 0.1% formic acid; B: acetonitrile containing 0.1% formic acid.

质谱条件:采用电喷雾电离源(ESI),正离子模式;离子源温度为300 ℃;毛细管温度为350 ℃;归一化碰撞能为35%;氮气作为鞘气及辅助气,其中鞘气压力为0.28 MPa,辅助气压力为0.07 MPa;氦气用作碰撞气;喷雾电压为4000 V;棱镜电压为85 V。在自动触发数据依赖采集模式下,采集了各分析物的MS^1^~MS^4^数据,选择MS^2^中丰度最高的离子为定量离子,在全扫描模式下,选择目标分析物的[M+H]^+^ *m/z*和保留时间监测其MS^2^离子。各分析物的多级质谱数据及相对丰度见表3。

**表3:** 各分析物的化学式、保留时间及离子对

Analyte	Molecular formula	Retention time/min	Ion pairs (*m/z*)
4CN-AB-BUTINACA	C_18_H_23_N_5_O_2_	8.32	342>325^*^>297, 342>297 (1.4%)
4F-AB-BUTINACA	C_17_H_23_FN_4_O_2_	9.22	335>318^*^>290, 335>290 (1.1%)
5F-AB-PINACA	C_18_H_25_FN_4_O_2_	9.86	349>332^*^>304, 349>304 (2.7%)
MDMB-INACA	C_15_H_19_N_3_O_3_	10.23	290>258^*^, 290>230 (78.9%)
AB-FUBINACA	C_20_H_21_FN_4_O_2_	10.26	369>352^*^>324, 369>324 (3.2%)
5F-ADB-PINACA	C_19_H_27_FN_4_O_2_	10.60	363>346^*^>318, 363>318 (0.63%)
PX-2	C_22_H_25_FN_4_O_2_	10.62	397>380^*^>352, 397>352 (7.8%)
5Cl-AB-PINACA	C_18_H_25_ClN_4_O_2_	10.65	365>348^*^>320, 365>320 (0.6%)
ADB-BINACA	C_21_H_24_N_4_O_2_	10.88	365>348^*^>320, 365>320 (0.3%)
ADB-FUBINACA	C_21_H_23_FN_4_O_2_	10.94	383>366^*^>338, 383>204 (16.4%)
ADB-BUTINACA	C_18_H_26_N_4_O_2_	11.07	331>314^*^>286>201, 331>286 (2.3%)
AB-PINACA	C_18_H_26_N_4_O_2_	11.07	331>314^*^>286>215, 331>286 (2.3%)
ADB-4en-PINACA	C_19_H_26_N_4_O_2_	11.19	343>326^*^>298, 343>298 (8.1%)
4CN-MDMB-BUTINACA	C_20_H_26_N_4_O_3_	11.75	371>311^*^, 371>339 (82.0%)
AB-CHMINACA	C_20_H_28_N_4_O_2_	11.83	357>340^*^>312, 357>312 (2.2%)
ADB-PINACA	C_19_H_28_N_4_O_2_	11.88	345>328^*^>300, 345>300 (1.9%)
4F-MMB-BUTINACA	C_18_H_24_FN_3_O_3_	11.94	350>290^*^, 350>318 (71.9%)
4CN-CUMYL-BUTINACA	C_22_H_24_N_4_O	12.06	361>243^*^>226, 361>226 (2.8%)
CUMYL-THPINACA	C_23_H_27_N_3_O_2_	12.40	378>260^*^>243, 378>243 (7.4%)
5F-MMB-PINACA	C_19_H_26_FN_3_O_3_	12.53	364>304^*^>233, 364>332 (0.3%)
ADB-CHMINACA	C_21_H_30_N_4_O_2_	12.60	371>354^*^>326, 371>326 (1.9%)
4F-MDMB-BUTINACA	C_19_H_26_FN_3_O_3_	12.70	364>304^*^, 364>332 (62.8%)
MMB-FUBINACA	C_21_H_22_FN_3_O_3_	12.83	384>324^*^, 384>352 (35.7%)
4F-CUMYL-BUTINACA	C_21_H_24_FN_3_O	12.93	354>236^*^>219, 354>219 (3.7%)
5F-MDMB-PINACA	C_20_H_28_FN_3_O_3_	13.18	378>318^*^, 378>346 (30.9%)
5F-EMB-PINACA	C_20_H_28_FN_3_O_3_	13.18	378>304^*^, 378>332 (78.9%)
5Cl-MMB-PINACA	C_19_H_26_ClN_3_O_3_	13.25	380>320^*^, 380>348 (50.6%)
5F-CUMYL-PINACA	C_22_H_26_FN_3_O	13.44	368>250^*^>233, 368>233 (2.9%)
MDMB-FUBINACA	C_22_H_24_FN_3_O_3_	13.48	398>338^*^, 398>366 (36.6%)
EMB-FUBINACA	C_22_H_24_FN_3_O_3_	13.48	398>324^*^, 398>352 (95.5%)
4CN-ABUTINACA	C_23_H_28_N_4_O	13.73	377>135^*^>107, 377>107 (2.2%)
5F-EDMB-PINACA	C_21_H_30_FN_3_O_3_	13.81	392>318^*^, 392>346 (85.4%)
5Cl-MDMB-PINACA	C_20_H_28_ClN_3_O_3_	13.88	394>334^*^, 394>362 (46.4%)
MDMB-BUTINACA	C_19_H_27_N_3_O_3_	13.89	346>286^*^, 346>314 (75.8%)
MDMB-4en-PINACA	C_20_H_27_N_3_O_3_	13.90	358>298^*^, 358>326(21.7%)
5F-MN-18	C_23_H_22_FN_3_O	13.91	376>233^*^>213, 376>251 (17.4%)
THJ-2201	C_23_H_21_FN_2_O	14.09	361>233^*^>213, 361>251(24.2%)
5Cl-CUMYL-PINACA	C_22_H_26_ClN_3_O	14.12	384>266^*^>249, 384>249 (3.0%)
5F-SDB-005	C_23_H_21_FN_2_O_2_	14.17	377>233^*^>213, 377>251 (26.8%)
4F-ABUTINACA	C_22_H_28_FN_3_O	14.69	370>135^*^>107, 370>107 (1.4%)
EDMB-PINACA	C_21_H_31_N_3_O_3_	15.16	374>328^*^, 374>300 (95.4%)
5F-APINACA	C_23_H_30_FN_3_O	15.22	384>135^*^>107, 384>367 (1.4%)
MDMB-CHMINACA	C_22_H_31_N_3_O_3_	15.28	386>326^*^, 386>354 (32.9%)
SDB-005	C_23_H_22_N_2_O_2_	15.35	359>215^*^>145, 359>233 (4.8%)
MN-18	C_23_H_23_N_3_O	15.35	358>215^*^>145, 358>233 (2.4%)
FUB-APINACA	C_25_H_26_FN_3_O	15.50	404>135^*^>107, 404>387 (15.9%)
APINACA-2H	C_23_H_31_N_3_O	15.72	366>135^*^>107, 366>189 (7.4%)
5F-APINAC	C_23_H_29_FN_2_O_2_	15.74	385>135^*^>107, 385>367 (0.3%)
5Cl-APINACA	C_23_H_30_ClN_3_O	15.90	400>135^*^>107, 400>383 (1.1%)
APINACA	C_23_H_31_N_3_O	16.92	366>135^*^>107, 366>107 (2.2%)
ACHMINACA	C_25_H_33_N_3_O	18.17	392>135^*^>107, 392>379 (1.5%)
APINACA-d_9_	C_23_H_22_D_9_N_3_O	16.77	375>135^*^

* Quantitative ion. The values in parentheses are the relative intensity of the corresponding ions.

## 2 结果与讨论

### 2.1 质谱条件的优化

根据表3,在51种SCs的MS^2^谱图中,4CN-AB-BUTINACA等27种SCs第二强碎片离子丰度比小于5%; PX-2等4种SCs第二强碎片离子丰度比为5%~10%; MDMB-INACA等20种SCs第二强碎片离子丰度比大于20%。为更加准确定性,利用离子阱质谱具备MS*^n^*优势,对于仅存在单个MS^2^碎片的化合物或MS^2^定性离子对丰度较弱的SCs,则采用MS^3^碎片离子进行确证。

通过对51种SCs多级质谱分析,发现5种不同结构类型的SCs具有如图2所示的质谱特征。如图2a所示,对含有取代乙酰胺基团的SCs,其MS^2^得到的基峰碎片离子均为[M+H-17]^+^,推测为丢失NH_3_中性分子而产生的碎片,相对于基峰离子,第2强碎片离子强度均较弱,除了ADB-FUBINACA离子丰度比为16.4%外,其余14种SCs丰度比均小于10%,因此考虑采用MS^3^离子作为定性离子,即[M+H-17]^+^碎片离子进行MS^3^分析时进一步丢失CO得到的[M+H-45]^+^碎片离子。与此同时,此类SCs中存在一对同分异构体,为ADB-BUTINACA和AB-PINACA,两个化合物的R_1_分别为正丁基和正戊基,R_2_分别为叔丁基和异丙基,这导致其MS^1^、MS^2^和MS^3^的质谱裂解均一致,且保留时间(11.07 min)相同。进行MS^4^分析时发现,ADB-BUTINACA和AB-PINACA分别产生碎片*m/z* 201和215,因此可用MS^4^对这两个化合物同时进行定性定量分析。

**图 2:**
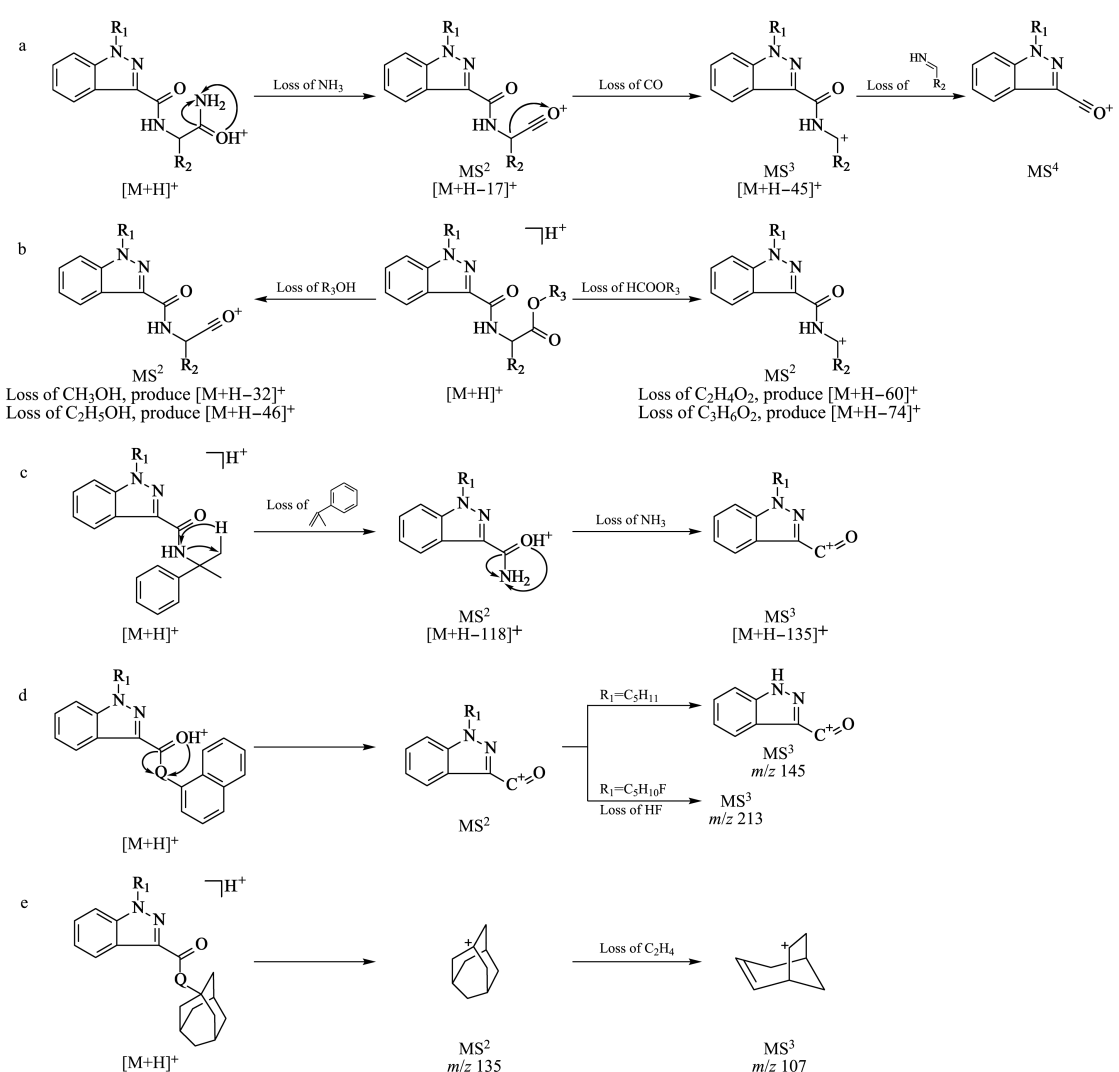
不同结构SCs的主要质谱断裂途径

如图2b所示,对含有取代乙酸甲酯基或取代乙酸乙酯基结构的SCs, MS^2^均得到至少2个丰度较强的碎片离子,分别为丢失甲酸甲酯(或甲酸乙酯)和甲醇(或乙醇)而产生的[M+H-HCOOR_3_]^+^和[M+H-R_3_OH]^+^碎片离子,其中R_3_为-CH_3_或-C_2_H_5_。此类化合物中存在3组同分异构体。其异构主要存在两种情况:第一,源于R_1_和R_2_的异构。化合物5F-MMB-PINACA和4F-MDMB-BUTINACA在丢失甲酸甲酯(*m/z* 364>304)和甲醇(*m/z* 364>332)后依旧具有相同的离子通道,但其保留时间分别为12.53和12.70 min,通过保留时间可将两者区分。第二,源于R_2_和R_3_的异构。化合物5F-MDMB-PINACA和5F-EMB-PINACA以及MDMB-FUBINACA和EMB-FUBINACA,两组同分异构体前者的R_2_和R_3_基团分别为叔丁基和甲基,而后者的R_2_和R_3_基团分别为异丙基和乙基。这导致在丢失甲酸甲酯(或甲酸乙酯)和甲醇(或乙醇)后产生了*m/z* 相差14的碎片离子,尽管具有相同的保留时间,但却具有不同的离子通道,可将其分开。

如图2c所示,对含有2-苯基-异丙基结构的SCs, MS^2^会优先丢失1-丙烯-2-苯基,产生[M+H-118]^+^的特征碎片,该碎片进一步丢失NH_3_产生MS^3^特征碎片[M+H-135]^+^。尽管[M+H-135]^+^这一特征碎片在MS^2^也存在,但其丰度比小于10%,采用这一碎片作为定性离子会极大地降低分析灵敏度。

如图2d所示,对含有萘基的SCs,当Q为氧原子时,MS^2^仅得到一个丢失1-萘酚而产生的碎片离子[M+H-144]^+^,根据R_1_结构的不同会呈现出不同的MS^3^碎片。当Q为NH(酰胺)或不存在(酮)时,则MS^2^得到2个离子,分别为[M+H-143]^+^和[M+H-125]^+^(Q=NH)、[M+H-128]^+^和[M+H- 110]^+^(Q=N/A)。

如图2e所示,对含有金刚烷基的SCs, MS^2^中均产生金刚烷基碎片离子*m/z* 135,该碎片离子进一步碎裂会产生*m/z* 107等碎片离子。其中存在同分异构体APINACA和APINACA-2H源于吲唑环1位和2位氮原子的取代,尽管具有相同的MS^2^基峰碎片*m/z* 135,但其保留时间分别为16.92和15.72 min,可以实现完全分离。

根据上述分析,采用多级质谱分析可以区分包含10个化合物的5组同分异构体,化合物ADB-BUTINACA和AB-PINACA需要采用MS^4^碎片进行结构确认。除此之外,其余化合物均可以采用MS^2^碎片离子和保留时间进行确认,选取其中响应最高的MS^2^碎片离子作为定量离子,以目标分析物[M+H]^+^的*m/z*和保留时间监测其MS^2^离子,记录51种SCs的MS^2^数据。具有特定质量的化合物在设定的采集时间窗口对MS^2^碎片进行自动监测,由于扫描过程中在特定时间范围内减少了需要监测的母离子,极大地提高了监测方法的灵敏度,其中每个化合物的监测时间区间设置为1 min,为化合物保留时间前后的±0.5 min。

### 2.2 色谱条件的优化

化合物在色谱分析中的保留行为是分析物与固定相和流动相相互作用的结果,因此选择合适的分析柱和流动相对化合物的分析具有重要意义。比较了相同规格的Phenyl-Hexyl(100 mm×2.1 mm, 1.8 μm)和ZORBAX Eclipse Plus C_18_(100 mm×2.1 mm, 1.8 μm)色谱柱的分离情况,以同分异构体5F-MMB-PINACA和4F-MDMB-BUTINACA为例,图3a和3b分别显示了两种化合物在Phenyl和C_18_色谱柱的分离情况,结果表明C_18_作为分析柱时,分析物具有更好的分离度、峰形和响应值。

**图 3:**
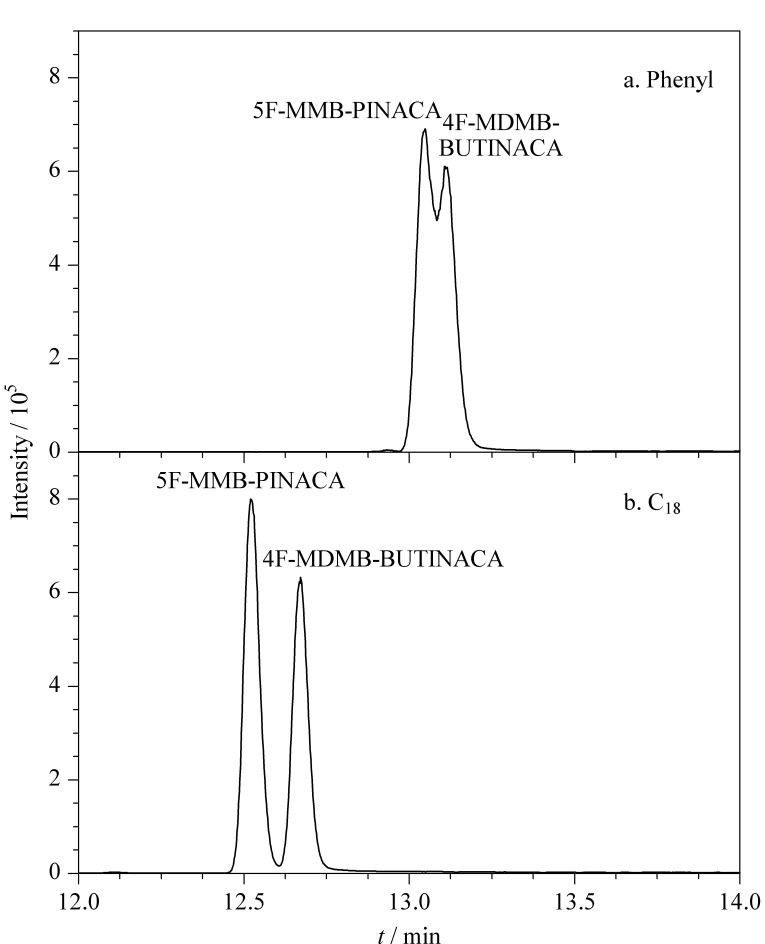
采用(a)苯基柱和(b)C_18_柱时5F-MMB-PINACA和4F-MDMB-BUTINACA的色谱图

在流动相的选择上,有机相考察了甲醇和乙腈,水相考察了纯水、0.1%甲酸溶液、10 mmol/L醋酸铵溶液和含0.1%甲酸的10 mmol/L醋酸铵溶液,结果见图4。其中根据[M+H]^+^峰的强度,乙腈的分离效果优于甲醇,乙腈相较于甲醇具有中等洗脱能力、强溶解能力以及低黏度等特点,因此其灵敏度更高,响应更强,并且柱压较小。以纯水为流动相时,大多数化合物的[M+H]^+^峰具有最高的响应值,但峰形较差,且产生较强的[M+Na]^+^峰,会对定量结果产生影响。以10 mmol/L醋酸铵溶液为流动相可有效抑制[M+Na]^+^峰的形成,并且改善了化合物的峰形。同时以含0.1%甲酸的10 mmol/L醋酸铵溶液为流动相时,甲酸可以提高离子化率,促进母离子[M+H]^+^峰的形成,使得[M+H]^+^峰的强度增大。综合考虑,最终选择了以含0.1%甲酸的乙腈-含0.1%甲酸的10 mmol/L醋酸铵溶液作为流动相对化合物进行检测。

**图 4:**
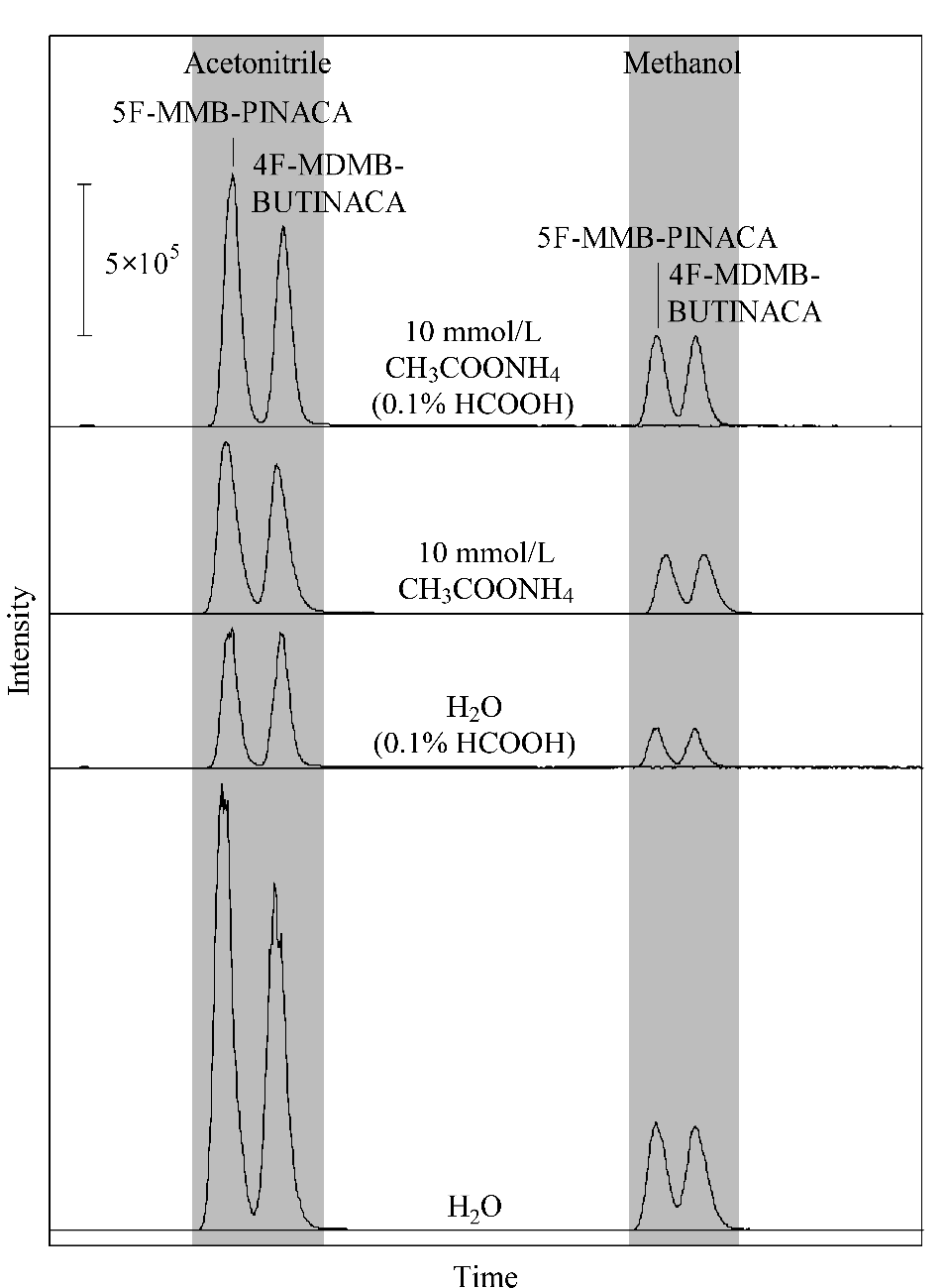
不同流动相下5F-MMB-PINACA和4F-MDMB-BUTINACA的色谱图

### 2.3 乙腈含量对萃取效果的影响

血液等生物样品采用SPE处理前,需要进行蛋白沉淀,常用的蛋白沉淀剂有乙腈、丙酮等有机溶剂^[38⇓⇓-41]^。乙腈、丙酮等溶剂在沉淀蛋白的同时,还可将目标分析物溶解到溶液中,利于SPE萃取。但是样品溶液中乙腈等有机溶剂的含量过高,会产生穿透效应,对SPE柱吸附及检测造成影响,因此需要在蛋白质沉淀处理后对样品进行稀释。为了确定稀释比例,本文通过对含不同体积分数乙腈的样品溶液进行测试,来确定最优的稀释剂加入量。移取0.1 mL质量浓度为500 ng/mL的混合标准溶液10份,分别加入0、0.1、0.2、0.3、0.4、0.5、0.6、0.7、0.8、0.9 mL乙腈后,用含0.1%甲酸的10 mmol/L醋酸铵溶液定容至1.0 mL,配制成乙腈含量为10%~100%的溶液并直接对其进行测试。结果表明,在不同乙腈体积分数下,所有SCs均能检测到。对于极性较强、保留时间较小的化合物,随着乙腈含量的增加,峰形变宽,当乙腈含量大于50%时出现了明显的峰拖尾现象,这是由于随着乙腈含量的增高,化合物随乙腈流动在SPE柱扩散,不利于SPE柱吸附,导致峰形变宽变差。此外,考察了不同乙腈含量下化合物的响应情况,如图5,采用相对峰面积表示化合物在10%~100%乙腈含量下的响应值,结果显示绝大多数化合物在40%乙腈含量下具有最高的响应。因此,对于1.3节中样品制备过程,在以乙腈沉淀蛋白后还需加入1.75 mL含0.1%甲酸(v/v)的醋酸铵溶液(10 mmol/L)稀释剂对样品进行稀释,以控制待测样品溶液中乙腈含量为40%,从而得到更好的响应。

**图 5:**
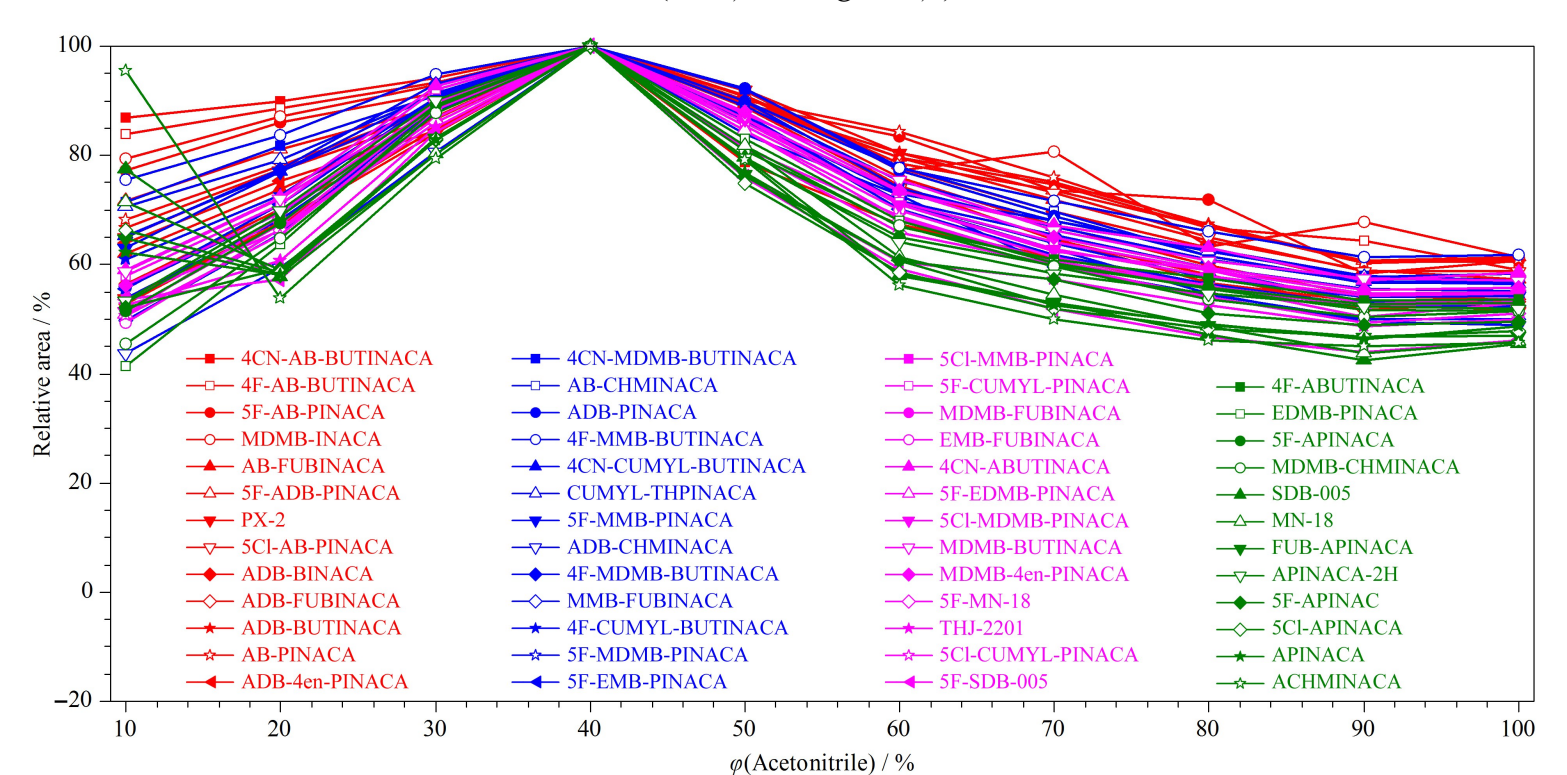
不同乙腈含量下SCs的相对峰面积

### 2.4 样品溶液稀释剂的选择

按照1.3节样品制备过程,将制备过程中的稀释剂分别替换为酸性(含0.1%甲酸的10 mmol/L醋酸铵溶液,pH=4.8)、中性(超纯水,pH=7.0)和碱性(三羟甲基氨基甲烷(Tris)溶液,pH=9.2)3种不同pH的稀释液,考察了稀释剂pH对目标化合物萃取的影响。结果表明,在3种pH下均能萃取到51种SCs,主要是实验所用的online SPE柱为Oasis HLB柱,其填料由亲水性的*N*-乙烯吡咯烷酮和亲脂性的二乙烯基苯聚合而成,是一种亲水亲脂平衡的可浸润反相吸附剂,可同时吸附酸性、中性和碱性化合物。图6显示了51种SCs在不同pH稀释液中的峰面积,在3种条件下响应差别不大,在中性和酸性条件下萃取效果较好,考虑到流动相为酸性,因此选择pH=4.8的含0.1%甲酸的10 mmol/L醋酸铵溶液为样品稀释液。

**图 6:**
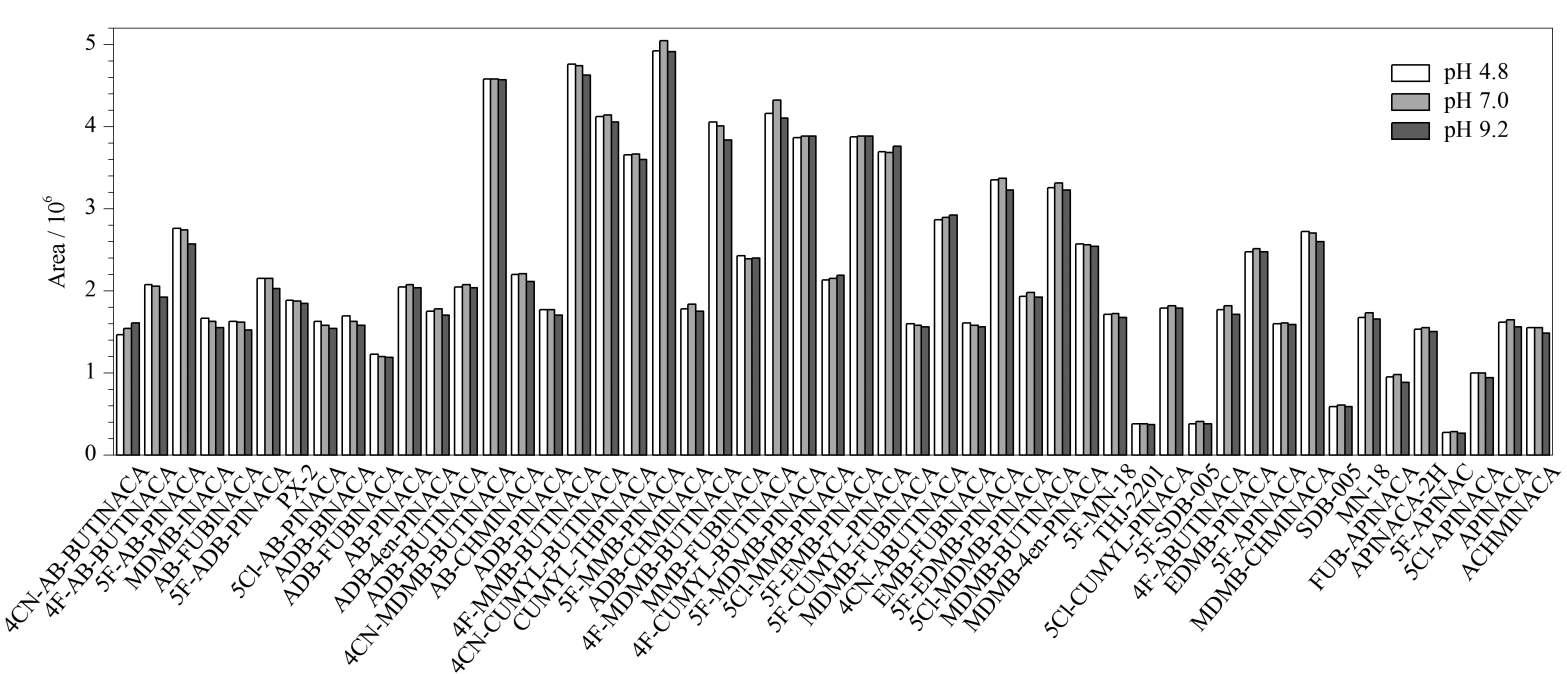
不同pH稀释液下各分析物的峰面积

### 2.5 检出限、定量限和线性范围

在空白尿液和血液中添加系列标准溶液,得到质量浓度分别为0.01、0.02、0.04、0.1、0.2、0.4、1、2、4、10、20、30、40、80、90和100 ng/mL的尿液和血液系列基质匹配标准溶液,按1.3节进行处理。以目标物在血液和尿液中的质量浓度为横坐标(*x*, ng/mL),目标物与内标的峰面积比值为纵坐标(*y*),采用最小二乘法进行回归,选择权重因子为1/*x*,在Xcalibur Quan Browser软件中进行了标准工作曲线拟合。以信噪比(*S/N*)≥3确定方法的检出限(LOD),以样品测定值的相对标准偏差(RSD)≤20%,并且准确度在±20%以内时方法所能检测到的分析物的最低含量作为定量限(LOQ), LOQ即为线性范围的下限。

表4显示了各分析物在尿液和血液中的LOD、线性范围、线性方程和相关系数(*R*^2^)。尿液中SCs的LOD为0.02~1 ng/mL,其中68%以上SCs的LOD ≤0.1 ng/mL; LOQ为0.04~4 ng/mL,以LOQ为线性范围的最低点,各化合物在相应范围内线性良好,*R*^2^为0.9932~0.9993,其中只有2个化合物的*R*^2^小于0.9950。

**表4:** SCs在尿液和血液中的LOD、线性范围、线性方程和相关系数

Analyte	Urine						Blood					
	LOD/ (ng/mL)	Linear range/ (ng/mL)		Linear equation	*R*^2^		LOD/ (ng/mL)	Linear range/ (ng/mL)		Linear equation		*R*^2^
4CN-AB-BUTINACA	0.4	1-	100	*y*=0.0608*x*-0.0041	0.9985		0.2	0.4-	100	*y*=0.0388*x*-0.0052		0.9990
4F-AB-BUTINACA	0.4	1-	100	*y*=0.0655*x*-0.0339	0.9989		0.4	1-	100	*y*=0.0501*x*-0.0303		0.9966
5F-AB-PINACA	0.4	1-	100	*y*=0.0592*x*-0.0128	0.9986		0.4	1-	100	*y*=0.0518*x*+0.1853		0.9963
MDMB-INACA	1	2-	80	*y*=0.0749*x*-0.0696	0.9958		0.2	1-	40	*y*=0.0627*x*-0.0319		0.9964
AB-FUBINACA	0.2	1-	80	*y*=0.0482*x*-0.0195	0.9987		0.4	1-	100	*y*=0.0471*x*-0.0008		0.9962
5F-ADB-PINACA	0.2	0.4-	80	*y*=0.0347*x*-0.0026	0.9963		0.4	1-	80	*y*=0.0358*x*+0.0037		0.994
PX-2	0.2	0.4-	100	*y*=0.0922*x*-0.0116	0.9987		0.1	1-	100	*y*=0.0944*x*+0.0175		0.9969
5Cl-AB-PINACA	0.4	1-	100	*y*=0.0554*x*-0.0150	0.9961		1	4-	100	*y*=0.0593*x*+0.0437		0.9959
ADB-BINACA	1	4-	100	*y*=0.0244*x*-0.0536	0.9955		1	2-	100	*y*=0.0276*x*-0.0271		0.9951
ADB-FUBINACA	0.4	1-	100	*y*=0.0237*x*-0.0089	0.9960		1	2-	100	*y*=0.0242*x*-0.0055		0.9960
ADB-BUTINACA	0.1	0.4-	40	*y*=0.0780*x*-0.0036	0.9951		0.1	0.2-	80	*y*=0.0688*x*+0.0037		0.9958
AB-PINACA	0.1	0.4-	40	*y*=0.0780*x*-0.0036	0.9951		0.1	0.2-	80	*y*=0.0688*x*+0.0037		0.9958
ADB-4en-PINACA	0.4	1-	100	*y*=0.0195*x*-0.0021	0.9955		0.4	1-	100	*y*=0.0177*x*+0.0009		0.9970
4CN-MDMB-BUTINACA	0.04	0.4-	100	*y*=0.6366*x*-0.0701	0.9974		0.02	0.2-	100	*y*=0.7464*x*-0.0395		0.9989
AB-CHMINACA	0.1	0.4-	100	*y*=0.0617*x*-0.0053	0.9983		0.04	0.2-	40	*y*=0.0798*x*+0.0021		0.996
ADB-PINACA	0.4	1-	40	*y*=0.0136*x*+0.0053	0.9932		0.4	1-	40	*y*=0.0134*x*+0.0144		0.9903
4F-MMB-BUTINACA	0.04	0.1-	100	*y*=1.1110*x*-0.0292	0.9984		0.04	0.2-	100	*y*=1.1321*x*-0.0572		0.9983
4CN-CUMYL-BUTINACA	0.04	0.1-	40	*y*=0.5143*x*-0.0158	0.9966		0.04	0.2-	100	*y*=0.4580*x*-0.0157		0.9961
CUMYL-THPINACA	0.1	0.2-	100	*y*=0.1605*x*+0.0039	0.9958		0.1	0.2-	100	*y*=0.1387*x*+0.0041		0.9933
5F-MMB-PINACA	0.04	0.1-	100	*y*=1.1653*x*-0.0371	0.9990		0.04	0.2-	100	*y*=1.2467*x*-0.0542		0.9981
ADB-CHMINACA	0.4	1-	80	*y*=0.0239*x*-0.0018	0.9957		0.4	1-	40	*y*=0.0307*x*+0.0032		0.9946
4F-MDMB-BUTINACA	0.04	0.1-	100	*y*=0.8691*x*-0.0237	0.9993		0.04	0.2-	100	*y*=0.8810*x*-0.0397		0.9988
MMB-FUBINACA	0.02	0.1-	100	*y*=0.8590*x*-0.0247	0.9988		0.02	0.2-	100	*y*=1.0823*x*-0.0531		0.9983
4F-CUMYL-BUTINACA	0.04	0.2-	100	*y*=0.2372*x*+0.0072	0.9976		0.02	0.2-	100	*y*=0.1428*x*+0.0002		0.9974
5F-MDMB-PINACA	0.04	0.1-	100	*y*=0.9169*x*-0.0010	0.9968		0.02	0.1-	100	*y*=0.9966*x*+0.0486		0.9962
5F-EMB-PINACA	0.02	0.04-	100	*y*=0.8774*x*-0.0098	0.9979		0.02	0.2-	100	*y*=0.9603*x*-0.0062		0.9969
5Cl-MMB-PINACA	0.04	0.2-	100	*y*=0.6880*x*+0.0292	0.9975		0.04	0.2-	100	*y*=0.7391*x*+0.0169		0.9965
5F-CUMYL-PINACA	0.04	0.4-	100	*y*=0.2960*x*-0.0194	0.9972		0.04	0.4-	100	*y*=0.2143*x*+0.0075		0.9953
MDMB-FUBINACA	0.04	0.1-	80	*y*=0.7709*x*-0.0219	0.9962		0.04	0.2-	40	*y*=0.9096*x*-0.0446		0.9970
EMB-FUBINACA	0.04	0.1-	80	*y*=0.6790*x*-0.0209	0.9976		0.04	0.2-	40	*y*=0.8287*x*-0.0331		0.9973
4CN-ABUTINACA	0.04	0.2-	100	*y*=0.5181*x*+0.0197	0.9976		0.02	0.2-	100	*y*=0.6322*x*-0.0393		0.9974
5F-EDMB-PINACA	0.04	0.2-	100	*y*=0.5716*x*+0.0262	0.9978		0.04	0.2-	100	*y*=0.6233*x*+0.0152		0.9961
5Cl-MDMB-PINACA	0.04	0.2-	100	*y*=0.7639*x*+0.0035	0.9982		0.02	0.2-	100	*y*=0.8016*x*+0.0032		0.9959
MDMB-BUTINACA	0.02	0.04-	100	*y*=0.7882*x*-0.0055	0.9967		0.04	0.2-	100	*y*=0.8075*x*-0.0178		0.9977
MDMB-4en-PINACA	0.02	0.04-	100	*y*=1.3750*x*-0.0117	0.9968		0.02	0.2-	100	*y*=1.4463*x*+0.0261		0.9957
5F-MN-18	0.04	0.1-	80	*y*=1.1690*x*-0.0370	0.9963		0.04	0.2-	100	*y*=1.1100*x*-0.0460		0.9971
THJ-2201	0.2	1-	100	*y*=0.3411*x*-0.0975	0.9977		0.1	1-	100	*y*=0.3746*x*-0.1636		0.9955
5Cl-CUMYL-PINACA	0.1	0.4-	40	*y*=0.2143*x*-0.0149	0.9964		0.1	0.2-	40	*y*=0.1727*x*-0.0065		0.9937
5F-SDB-005	0.1	0.4-	100	*y*=0.1184*x*+0.0039	0.9965		0.1	0.2-	100	*y*=0.1390*x*-0.0068		0.9970
4F-ABUTINACA	0.02	0.4-	100	*y*=0.4280*x*-0.0284	0.9989		0.02	0.2-	100	*y*=0.4201*x*-0.0210		0.9983
EDMB-PINACA	0.04	0.2-	100	*y*=0.6040*x*+0.0257	0.9968		0.02	0.2-	100	*y*=0.6436*x*-0.0040		0.9958
5F-APINACA	0.04	0.2-	100	*y*=0.3369*x*+0.0143	0.9974		0.04	0.2-	100	*y*=0.3707*x*-0.0004		0.9960
MDMB-CHMINACA	0.04	0.1-	100	*y*=1.0240*x*-0.0188	0.9986		0.02	0.2-	100	*y*=1.1423*x*-0.0111		0.9961
SDB-005	0.1	0.2-	40	*y*=0.1540*x*+0.0156	0.9948		0.04	0.2-	40	*y*=0.1874*x*+0.0125		0.9938
MN-18	0.2	1-	100	*y*=0.9463*x*-0.2444	0.9954		0.1	0.2-	100	*y*=1.0564*x*-0.0026		0.9975
FUB-APINACA	0.04	0.4-	100	*y*=0.2070*x*-0.0032	0.9977		0.04	0.2-	100	*y*=0.2159*x*-0.0045		0.9976
APINACA-2H	0.2	1-	100	*y*=0.3442*x*-0.0990	0.9985		0.1	0.4-	100	*y*=0.5830*x*+0.0326		0.9954
5F-APINAC	0.1	0.4-	100	*y*=0.0516*x*+0.0021	0.9956		0.1	0.4-	80	*y*=0.0528*x*+0.0043		0.9950
5Cl-APINACA	0.1	0.4-	100	*y*=0.2084*x*-0.0116	0.9985		0.04	0.2-	100	*y*=0.2279*x*-0.0098		0.9972
APINACA	0.02	0.04-	100	*y*=0.3452*x*+0.0031	0.9989		0.02	0.2-	100	*y*=0.3491*x*-0.0070		0.9992
ACHMINACA	0.1	0.4-	100	*y*=0.2125*x*+0.0019	0.9993		0.04	0.2-	100	*y*=0.2051*x*-0.0036		0.9990

*y*: ratio of peak areas of the analyte to internal standard; *x*: mass concentration, ng/mL.

血液中SCs的LOD为0.02~1 ng/mL,其中76%以上SCs的LOD≤0.1 ng/mL; LOQ为0.1~4 ng/mL,以LOQ为线性范围的最低点,各化合物在相应范围内线性良好,*R*^2^为0.9903~0.9992,其中只有6个化合物的*R*^2^小于0.9950。

### 2.6 回收率和精密度

在空白血液和尿液中分别添加不同水平的混合标准溶液,配制成LOQ、低浓度(2倍LOQ)、中浓度(10 ng/mL)和高浓度(线性范围上限)4个水平的加标溶液,每个水平平行测定6次,考察方法的加标回收率和精密度(RSD)。

图7显示了各分析物在不同水平下的回收率和RSD分布图。尿液中,在LOQ水平下,方法的回收率为84.95%~115.71%, RSD为2.29%~13.40%;低、中、高水平下的回收率为86.63%~110.57%, RSD为0.72%~11.04%。血液中,在LOQ水平下,方法的回收率为83.47%~116.95%, RSD为2.44%~13.20%;低、中、高水平下的回收率为88.02%~113.38%, RSD为0.58%~13.79%。该方法回收率和精密度良好,能够满足SCs的定性和定量分析。

**图 7:**
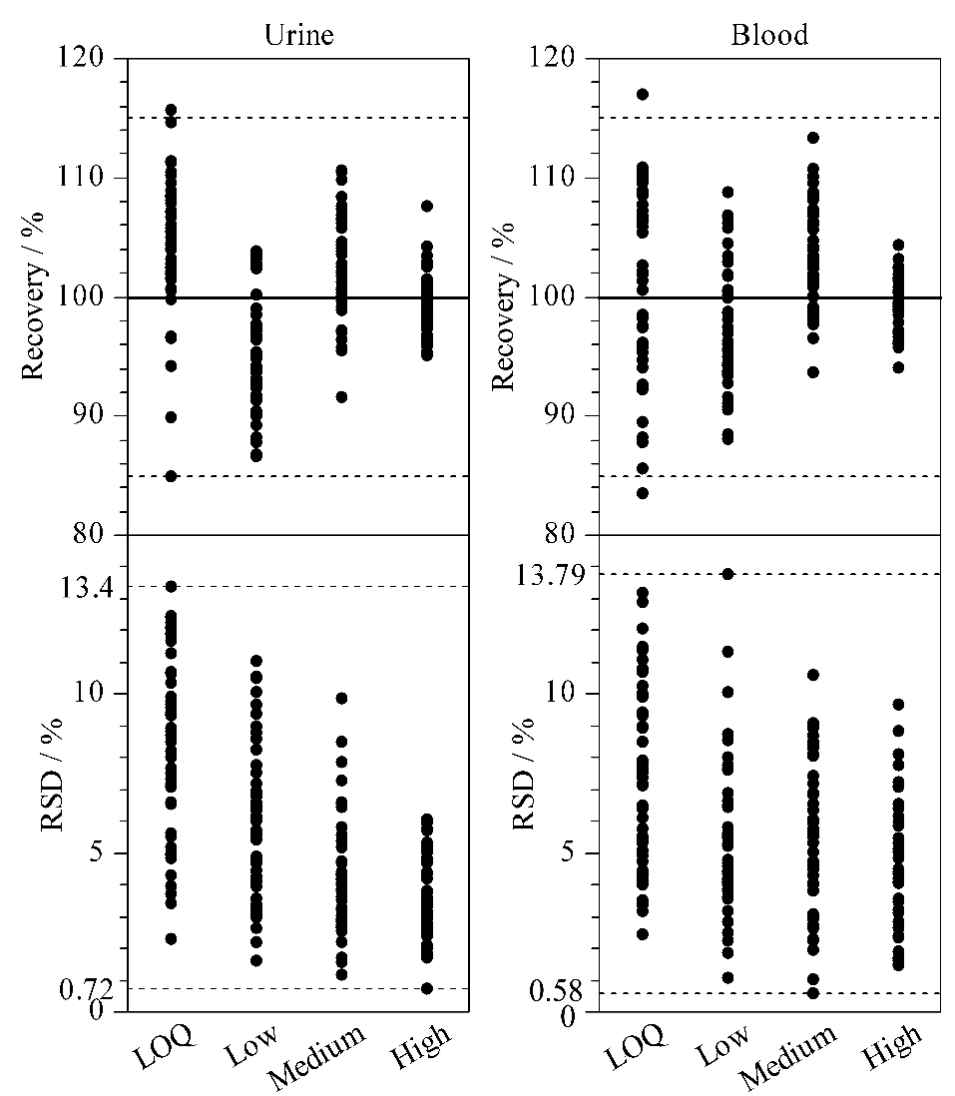
血液和尿液中各分析物的加标回收率和RSD分布(*n*=6)

### 2.7 与文献已有方法的对比

将本方法与相关文献报道的血液和尿液中SCs及其代谢物的分析检测方法进行了对比,见表5。在方法的前处理方式上,沉淀蛋白的处理方式通常无法去除生物基质中的盐;传统的SPE和液液萃取(LLE)的样品处理方法通常需要对目标分析物进行提取、氮吹和复溶等操作才进行后续的LC-MS分析。而本文使用的online SPE,样品经过简单沉淀蛋白后即可在仪器上实现样品的富集,并进入后端的LC-MS分析,极大地提高了分析效率,适合对大量样品进行快速筛查。同时,一种分析方法要进行准确定量分析的前提是能够准确甄别不同的SCs。本文方法使用的LIT-MS虽在定量分析上不具备明显的优势,但在定性分析方面,则能够有效利用LIT-MS的多级质谱数据区分51种吲唑类SCs中5组共10个同分异构体。因此,本文采用online SPE-LC-LIT-MS对血液和尿液中吲唑类SCs进行筛查和定性分析,能够满足样品的快速自动化筛查,得出准确的定量和定性数据,具有很高的实际应用价值。

**表5:** 本方法与其他方法的比较

Matrix	Number of analytes	Number of isomers	Pre-treatment methods	Analytical method	LOD/ (ng/mL)		LOQ/ (ng/mL)		Ref.
Urine	51	5 groups, 10	PPT/online SPE	LC-LIT-MS	0.02-	1	0.04-	4	this study
	17 metabolites	0	LLE	LC-MS/MS	0.01-	0.5	0.1-	0.5	[31]
	6	0	LLE	LC-MS/MS	0.003-	0.008	0.01		[45]
	11	0	SPE	LC-MS/MS	/		0.01-	0.1	[17]
Blood	51	5 groups, 10	PPT/online SPE	LC-LIT-MS	0.02-	1	0.1-	4	this study
	72	8 groups, 18	PPT	LC-MS/MS	0.01-	0.48	/		[29]
	23	1 group, 2	PPT	DART-MS/MS	0.01-	1	10		[46]
	6	0	PPT/SPE	UPLC-MS/MS	0.01-	0.05	0.05-	0.1	[30]
	15	1 group, 2	LLE	LC-MS/MS	0.025-	0.01	0.05-	0.1	[31]
	10	0	QuEChERS	UPLC-QE-Orbitrap-MS	0.01-	0.05	0.05-	0.20	[24]

PPT: protein precipitation; LIT: linear ion trap; LLE: liquid-liquid extraction; DART: direct analysis in real-time; UPLC-QE-Orbitrap-MS: ultra performance liquid chromatography-quadrupole exactive-orbitrap mass spectrometry; /: no data.

## 3 结论

本文基于online SPE-LC-LIT-MS,建立了快速检测血液和尿液中51种吲唑类SCs的分析方法,采用多级质谱和保留时间可以区分包含10个化合物的5组同分异构体。该方法实现了51种SCs的快速筛查和定量分析,并且回收率和精密度良好,能够满足SCs的定性和定量分析,可为相关案件中SCs的分析检测提供参考。
